# Morphological Diversity and the Roles of Contingency, Chance and Determinism in African Cichlid Radiations

**DOI:** 10.1371/journal.pone.0004740

**Published:** 2009-03-09

**Authors:** Kyle A. Young, Jos Snoeks, Ole Seehausen

**Affiliations:** 1 Department of Aquatic Ecology & Evolution, Institute of Ecology & Evolution, University of Bern, Bern, Switzerland; 2 EAWAG, Centre of Ecology, Evolution & Biogeochemistry, Kastanienbaum, Switzerland; 3 Zoology Department, Royal Museum for Central Africa, Tervuren, Belgium; 4 Department of Biology, University of Leuven, Leuven, Belgium; Duke University, United States of America

## Abstract

**Background:**

Deterministic evolution, phylogenetic contingency and evolutionary chance each can influence patterns of morphological diversification during adaptive radiation. In comparative studies of replicate radiations, convergence in a common morphospace implicates determinism, whereas non-convergence suggests the importance of contingency or chance.

**Methodology/Principal Findings:**

The endemic cichlid fish assemblages of the three African great lakes have evolved similar sets of ecomorphs but show evidence of non-convergence when compared in a common morphospace, suggesting the importance of contingency and/or chance. We then analyzed the morphological diversity of each assemblage independently and compared their axes of diversification in the unconstrained global morphospace. We find that despite differences in phylogenetic composition, invasion history, and ecological setting, the three assemblages are diversifying along parallel axes through morphospace and have nearly identical variance-covariance structures among morphological elements.

**Conclusions/Significance:**

By demonstrating that replicate adaptive radiations are diverging along parallel axes, we have shown that non-convergence in the common morphospace is associated with convergence in the global morphospace. Applying these complimentary analyses to future comparative studies will improve our understanding of the relationship between morphological convergence and non-convergence, and the roles of contingency, chance and determinism in driving morphological diversification.

## Introduction

Adaptive radiations are important sources of biodiversity, yet uncertainty persists over the degree to which such diversity results from deterministic evolution, phylogenetic contingency, and the chance ascension of different ridges in the adaptive landscape. Though relevant microevolutionary hypotheses can be tested experimentally, the macroevolutionary process of adaptive radiation in nature is best studied by comparing patterns of morphological diversity among replicate radiations of related lineages diversifying in similar environments [1]. Morphological convergence among radiations suggests deterministic evolution is strong enough a force to overcome variation in phylogenetic background and ecological setting that may differentially constrain the morphological ‘space’ available to diversifying lineages [1]–[4]. Alternatively, radiations from similar environments that are morphologically non-convergent provide evidence that contingency and/or evolutionary chance outweigh the effect of deterministic evolution [5]–[7].

Examples of convergent adaptive radiations in nature include fish from post-glacial lakes, frogs and mammals from different continents, and lizards and spiders from oceanic islands [2], [3], [8]–[10]. However, evolutionary communities from similar environments are often non-convergent [6], [11], [12]. Similarly, experimental studies using micro-organisms suggest adaptive radiation is often deterministic and convergent [13], [14], but also that replicate lineages diversifying in identical [6], [7] or similar [15] environments can be non-convergent due to evolutionary chance. Here we help resolve this empirical discord using the cichlid fish assemblages from the African great lakes.

The endemic cichlid fish assemblages of Lakes Victoria (LV, ≥450 sp.), Malawi (LM, ≥450 sp.) and Tanganyika (LT, ≥200 sp.) are the most speciose and ecologically diverse radiations known and uniquely suited for a comparative study of adaptive radiation. The fish communities of all three lakes are dominated by endemic assemblages that display qualitatively convergent sets of ‘ecomorphs’ occupying nearly every imaginable niche [16]–[18]. The oldest assemblage from LT is phylogenetically structured into several distinct clades which may have been seeded by multiple distantly related colonists [19]. The assemblages of LV and LM each have just one radiation of closely related species, which may have been seeded by multiple, albeit more closely related colonists [20]. The assemblages differ in age by up to three orders of magnitude (LV, 0.015–0.2 myr.; LM, 2–4 myr.; LT, 8–16 myr.) [19], providing rare comparative insight into the temporal progress of adaptive radiation. For a single exception [10], previous comparative studies provide limited insight into how morphological and taxonomic diversity accumulate through time because they have compared replicate adaptive radiations of similar [2] or uniformly old age [3], [6], [8], [9]. The three great lake cichlid assemblages are the evolutionary equivalent of three Galapagos archipelagos of widely different age, each with hundreds of endemic species with the niches and morphologies not only of finches, but of raptors, water fowl, and gulls.

We first show that when compared in a common morphospace the assemblages of endemic cichlid fishes from the three lakes show evidence of non-convergence. We then analyze patterns of morphological diversity for each assemblage independently and find that the assemblages are diversifying along common axes through the global morphospace. Together these analyses help resolve an apparent empirical discord between of convergence and non-convergence and offer a promising approach for improving our ability to determine the roles of chance, contingency and determinism in adaptive radiation.

## Methods

### Specimen collection and geometric morphometric analysis

**Figure 1:**
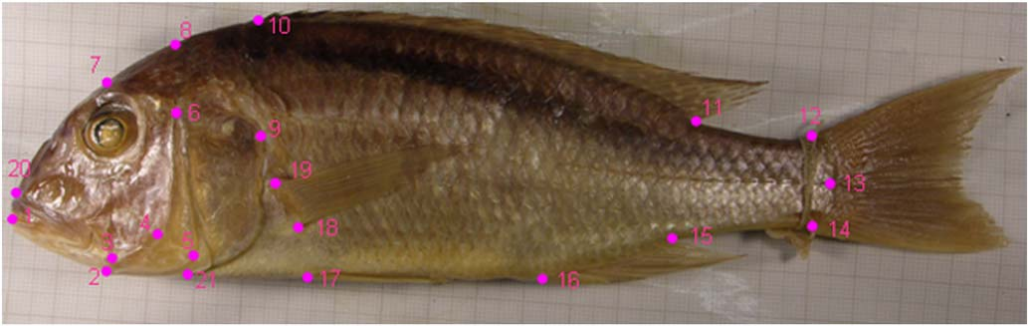
Locations of landmarks used in morphometric analyses. (1) anterior tip of lower jaw, (2) posterior tip of lower jaw , (3) posterior hinge of lower jaw, (4) ventral-posterior extreme of mandible plate, (5) ventral-posterior extreme of preopercle, (6) dorsal end of preopercle just below the pterotics, (7) dorsal margin of the head directly above the centre of the eye, (8) dorsal margin of the head directly above (6), (9) posterior extreme of gill-cover at opercular blotch, (10) anterior insertion of dorsal fin, (11) posterior insertion of dorsal fin, (12) dorsal insertion of caudal fin, (13) caudal border of hypural plate at the lateral line, (14) ventral insertion of caudal fin, (15) posterior insertion of anal fin, (16) anterior insertion of anal fin, (17) anterior/dorsal insertion of pelvic fin, (18) ventral insertion of pectoral fin, (19) dorsal insertion of pectoral fine, (20) anterior extreme of snout bone, (21) end of opercular membrane ventrally.

We collected digital images of the left side of representative individuals from the collections of the Natural History Museum (London, U.K.), Africa Museum (Tervuren, Belgium), Naturalis Museum (Leiden, Netherlands) and the personal collection of O.S. Importantly, Lake Victoria cichlids were sampled from collections made prior to wide spread extinctions associated with eutrophication and population expansion of introduced Nile perch (*Lates niloticus*) [21], [22]. For 125 individuals representing the taxonomic and morphological diversity of each assemblage (supplementary material, Table S1) we recorded the *x-y* coordinates of 21 landmarks using tpsDig 1.40 [23] (Figure 1).

We used partial warp analysis in tpsRelw version 1.42 [24] to quantify variation in shape while controlling for variation in size. The analysis scales landmarks of each specimen to a common body size, rotates each individual to a common alignment, then computes the average shape of all individuals included in the analysis to create a consensus shape. The partial warps describe the amount of stretching, bending and twisting necessary to superimpose the coordinates of all specimens onto the consensus shape. Each specimen has a weight for the *x*- and *y* components of each partial warp, with larger weight values associated with larger deviations from the consensus morphology (i.e. more extreme morphologies). The matrices of these partial warp weights are used for subsequent analyses. We do not control for phylogenetic independence in our analyses because species level phylogenies are not available for the Lake Malawi and Lake Victoria assemblages.

### Comparing morphological diversity in the common morphospace

We first followed the traditional comparative approach by including all 375 specimens in morphometric analyses of total shape (landmarks 1–21), body shape (9–19), head shape (2,7–9,20, 21), and jaw shape (1–6). We used tpsRelw to conduct principal components analysis (PCA) on the matrix of partial warp weights to yield relative warp scores, the equivalent of PCA scores for geometric morphometric data. From each analysis we retained the four relative warp axes that explained more than 5% of the variation in morphology. These axes are hereafter referred to as **M_max_-M_4_**.

**Figure 2:**
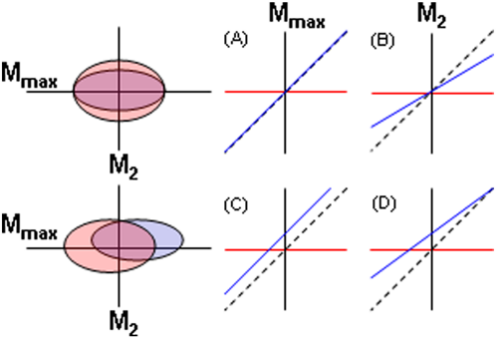
Comparing morphological diversity using ordered-axis plots. Ordered-axis plots discriminate between different patterns of morphological diversity along axes of a multidimensional morphospace. In this example species of two adaptive radiations with the same number of observations are represented by clouds of red (older, more diverse) and blue (younger, less diverse) points in two dimensional morphospaces defined by M_max_ and M_2_. When their values along an axis are independently ordered from smallest to largest then combined to form a set of *x*-*y* points, the slope and intercept (int.) of the linear regression of *y* (blue) on *x* (red) discriminate between four possible arrangements along that axis. The dotted line is slope = 1. Top: (A) along M_max_ the radiations are centered at the same point (i.e. convergent) so the int. = 0, and equally diverse, so the slope = 1; (B) along M_2_ the radiations are again centered at the same point (convergent, int. = 0) but the older radiation is more diverse so the slope of the regression of *y* on *x* is <1. Bottom: (C) along M_max_ the radiations are centered at different points along the axis (non-convergent, int.≠0) but have equal levels of diversity (slope = 1); (D) along M_2_ the radiations are non-convergent (int.≠0) and the older radiation is more diverse (slope<1).

We compared patterns of diversity in the common morphospaces using a new approach we call the ‘ordered-axis plot’. Ordered-axis plots are constructed and analyzed as follows (Figure 2). Along an axis of the common morphospace, the relative warp scores of two assemblages are first independently ordered from lowest to highest, then combined to create a set of 125 *x*-*y* points, with the older and more diverse assemblage-that with the larger range in values-placed on the *x*-axis. When these 125 points are plotted in a two dimensional *x*-*y* space, the intercept and slope of a simple linear regression of *y* on *x* statistically distinguish between the four possible arrangements of the two assemblages along that axis of the morphospace. If diversification during evolutionary radiation is convergent and rapid, the assemblages will be centered at the same point along the axis (intercept = 0) and be equally diverse (slope = 1) (Figure 2A). The assemblages may be similarly centered (int. = 0) but have age-ordered levels of diversity (slope<1) (Figure 2B), implying the assemblages are convergent but that morphological diversity along that axis accumulates more gradually. Alternatively, the assemblages may be equally diverse (slope = 1) but centered at different locations along an axis (int.≠0) (Figure 2C), suggesting diversification is rapid but non-convergent. Finally, if diversification is non-convergent and gradual, the assemblages will be centered at different locations along the axis (int.≠0) and have age-ordered levels of diversity (slope<1) (Figure 2D).

Ordered-axis plots have two advantages over using means and variances to test for differences in location and diversity, respectively, along axes of a common morphospace. First, they compare relative location and diversity using a single analysis associated with a simple visual representation. Second, the intercept and slope of ordered-axis plot regressions are more sensitive to extreme morphologies than tests for the equality of means and variances. Because our samples, like the assemblages themselves, are dominated by average rather than extreme phenotypes, this sensitivity is particularly important for testing whether assemblages are centered at different locations and have different levels of morphological diversity along different axes of morphospace. For comparison, for each of the 16 axes analyzed using ordered-axis plots we present the results of pairwise parametric tests for equal means (*t*-tests) and variances (*F* ratios) based on a table wide α = 0.003 = 0.05/16.

In all our analyses the 125 values from LT, which is the oldest and most morphologically diverse assemblage, are placed on the *x*-axis. The 125 values from LM and LV are placed on the *y*-axis. Thus, the regression lines of our ordered-axis plots represent the relationship between morphological variation among species from LM and LV (*y* values) versus variation among those of LT (*x* value). We discriminate between the four arrangements described above along **M_max_-M_4_** using the 99.99% confidence intervals for the slopes and intercepts from 10,000 bootstrapped linear regressions of LM and LV on LT (Note the analysis does not require that assemblages have equal numbers of species, only that the same number are randomly sampled from each). We use this strict significance level because of the number tests and the sensitivity of the analysis. For graphical clarity we test for differences between LV and LM by comparing their confidence intervals from regressions on LT. The results are the same as regressing LV on LM and testing for intercept = 0 and slope = 1.

### Comparing morphological diversity in the global morphospace

Comparing patterns of diversity in a common morphospace requires that the diversities of the assemblages are summarized along common axes, even if the true axes of diversification actually vary among assemblages. We removed this constraint by analyzing each assemblage separately, which allows the axes of morphological divergence to be defined independently for each assemblage. We then compared these axes of diversification to test whether the three assemblages are diversifying in parallel through the unconstrained global morphospace.

We recalculated the same four partial warp matrices (total, body, head and jaw shape) for each assemblage separately and conducted PCA on each partial warp matrix to yield relative warp scores. For each assemblage we again retained **M_max_-M_4_** (those axes explaining >5% of variation) for total shape and its three elements. We then tested whether the assemblages are diverging in parallel through the global morphospace using SpaceAngle6b [25], [26]. Formally, this tests whether the angles between two assemblages’ **M_max_** axes, 2-D planes, and 3- and 4-D spaces of morphological divergence are more different than the angles between two random samples of either single assemblage. First, we calculated the 95% confidence interval (CI) for the angle between two assemblages by re-sampling 100 specimens of each assemblage with replacement and calculating the angle between them 700 times. We then tested the null hypothesis that the angle between two assemblages could result from the random subdivision of either assemblage, i.e. that the assemblages are ‘the same’. Each assemblage was randomly partitioned into two and 4900 bootstrapped angles between them calculated. Two assemblages were considered to be diversifying along non-parallel axes if the lower 95% CI for the between assemblage angle was greater than the larger of the two upper 95% CI for within assemblage estimates. For all analyses we used the maximum sample sizes and replicates allowed by the software.

### Comparing morphological variance-covariance structures

The results of global morphospace analysis suggest that body, head and jaw shape covary similarly in the three assemblages. To formally test the hypothesis that the different elements of total shape covary similarly we further decomposed body, head and jaw shape into three, two and two sub-elements, respectively [upper body (9–11), caudal area (11–15), lower body (16–18), upper head (7–9), lower head (9,20,21), cheek (3–6), and lower jaw (1–3)(Figure 1)]. Importantly, no two sub-elements share more than a single landmark, so variation in the shape of one does not strictly require or affect variation in the other. For each assemblage we conducted PCA in tpsRelw on the partial warp matrix for each of the seven sub-elements separately. For each sub-element we first confirmed that the **M_max_** scores of each assemblage had the same relationship between sign (positive and negative) and shape change. For example, we checked that for the lower jaw triangle (points 1–3 in Figure 1) positive **M_max_** scores corresponded to lengthening in each assemblage (in cases where they were reversed, we multiplied all values by −1). For each assemblage we then used the 125 specimens’ seven **M_max_** scores to calculate a 7×7 morphological variance-covariance matrix. We tested whether shape across the seven independent sub-elements covaried similarly by comparing the variance-covariance matrices using Mantel’s test of matrix correlation in MANTEL version 1.15 [27] with 10,000 random row permutations of one of the matrices.

## Results

### Morphological diversity in the common morphospace

**Figure 3:**
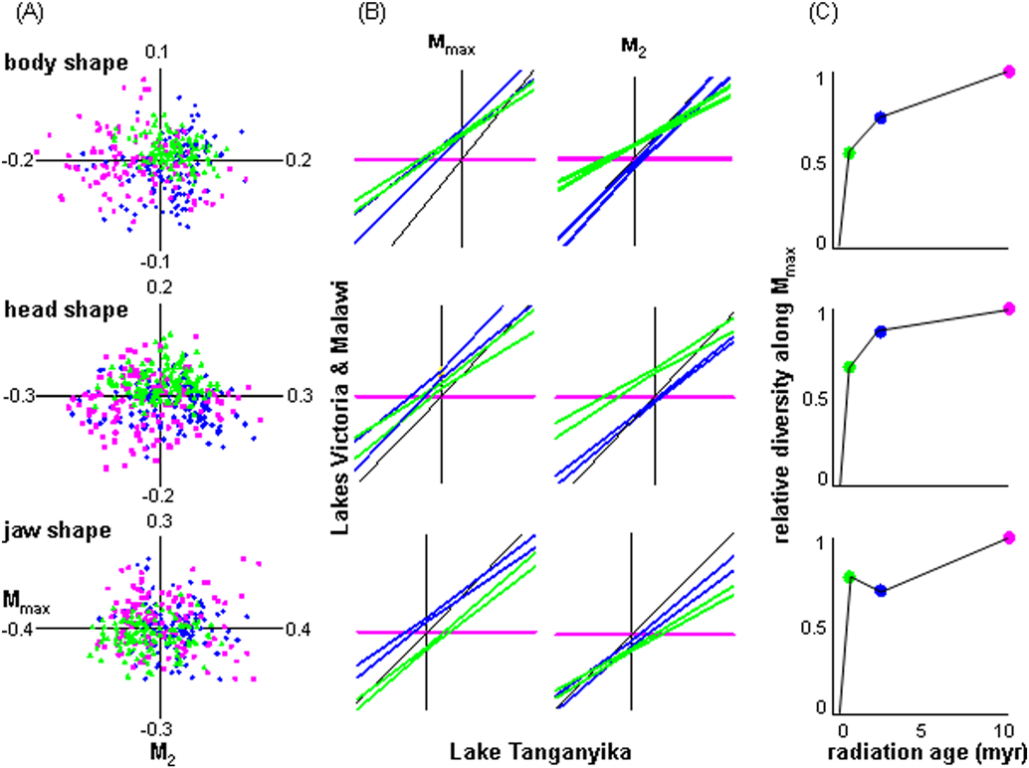
Morphological diversity in the common morphospace. Variation in body, head and jaw shape diversity in common morphospaces among the cichlid assemblages from Lakes Victoria (LV-green), Malawi (LM-blue) and Tanganyika (LT-pink). (A) Locations of species of the three assemblages in the three morphospaces. (B) Ordered-axis plots along M_max_ and M_2_ (with LT along the *x*-axis) showing the 99.99% confidence intervals of linear regressions of LV and LM on LT. Non-equal intercepts show assemblages are centered at different locations along the axis. Non-equal slopes indicate the assemblages have different levels of diversity along the axis. See Table 1 for tests of equality for the intercepts and slopes. (C) The relationship between assemblage age and relative morphological diversity (slopes of the regression of LV and LM on LT from the ordered-axis plots, with LT = 1 ) along the three M_max_ axes. For these plots the approximate ages of the assemblages are: LV-0.1 myr., LM-2 myr., LT-10 myr. Note that the lines connecting the points are included for comparison, not to imply temporal trends in diversity of a single radiation.

The common morphospace analysis provides three insights (Figure 3, Table 1). First, despite occupying broadly overlapping regions of morphospace, the cichlid assemblages from the three African great lakes show consistent evidence of non-convergence. Figure 3A shows for each of the three elements of total shape the locations of the 375 individuals in the two dimensional morphospaces defined by **M_max_** and **M_2_**. The non-zero intercepts of the ordered-axis plots of LM and LV (*y*-axis) regressed on LT (*x*-axis) suggest that only along **M_max_** for body shape are two assemblages centered at the same point in morphospace (Figure 3B, Table 1). For total shape and its three elements, along no axis explaining ≥5% of morphological diversity (**M_max_-M_4_**) are all three assemblages centered at the same location; along 12 of 16 axes the three assemblages are each centered at a different location (Table 1). Standard *t*-tests detect fewer significant differences in locations along the axes. For example, for total shape and its three elements, along five axes all assemblages have the same mean, while along only three axes do all three assemblages have different means.

**Table 1:** Comparison of cichlid assemblages in the common morphospace.

axis	total shape			body shape			head shape			jaw shape		
	%	slope	int.	%	slope	int.	%	slope	int.	%	slope	int.
**M_max_**	32	v<m<1 (v<m = t)	0<m = v (t<m = v)	47	v<m<1 (v<m = t)	0<v = m (t<v = m)	49	v<m = 1 (v<m = t)	0<v<m (t<v = m)	53	m = v<1 (m<v = t)	v<0<m (v<t = m)
**M_2_**	21	v = m = 1 (v = m = t)	v<0<m (v<t = m)	15	v<m = 1 (v<m = t)	m<0<v (m = t<v)	17	v<m<1 (v<m = t)	m<0<v (m = t<v)	26	v<m<1 (v<m = t)	v<m<0 (v = m = t)
**M_3_**	13	m<v<1 (m<v = t)	0<m = v (t<m = v)	11	v<m<1 (v = m<t)	m<v<0 (m = v = t)	16	v = m<1 (v = m = t)	0<m<v (t = m = v)	10	v<m = 1 (v<m = t)	m<v<0 (m = v = t)
**M_4_**	5	v<m<1 (v<m = t)	v<0<m (v<t<m)	8	v = m<1 (v = m<t)	m = v<0 (m = v<t)	7	v = m,m = 1 (v = m = t)	0<v<m (t = v = m)	6	v = m<1 (v = m = t)	v<0<m (v<t<m)

The results of ordered-axis plot comparisons along the first four axes (**M_max_-M_4_**) of total shape and its three elements. The percent of the total variance explained by each axis is presented in the first column for each analysis. The axes scores of species from Lakes Malawi (m) and Victoria (v) are regressed on those from Lake Tanganyika (defined as slope = 1, intercept = 0). Assemblages with equal intercepts are centered similarly along the axis. Assemblages with equal slopes are equally diverse along the axis. When statistically equal, the assemblages are in rank order, left to right. Equality is tested using the 99.99% confidence intervals for the slopes and intercepts from 10,000 bootstrapped linear regressions of LM and LV on LT. In parentheses below the results of ordered-axis plot regressions are results of *F* tests for equal variances (equal slopes) and *t*-tests for equal means (equal intercept) based on a table wide *P* = 0.003 = 0.05/16.

Second, morphological diversity appears to accumulate continually and be unrelated to species richness. For all but three of the16 axes the rank order of diversity (i.e. slopes of LM and LV on LT with LT = 1) matches that of assemblage age (Figure 3, Table 1). For example, the slopes of the ordered-axis plots (Figure 3B) show that along **M_max_** and **M_2_** for the three elements of total shape, LV is never as diverse as LT (all slopes<1) and is as diverse as LM only along **M_max_** for jaw shape. Species richness is higher in the young and middle-aged LV and LM assemblages, respectively, than in the older and morphologically more diverse LT assemblage. As before, *F* ratio tests for the equality of variances revealed similar patterns with fewer significant differences.

Finally, morphological diversity in head and jaw shape appears to accumulate faster than in body shape. Whereas shape diversity is age-ordered along **M_max_**-body, LV is as diverse as LM along **M_max_**-jaw and LM as diverse as LT along **M_max_**-head (Figures 3B,C). These observations regarding the temporal accumulation of different components of morphological diversity hold regardless of the exact ages of the three assemblages, the reasonable estimates of which do not overlap [19]–[20].

### Morphological diversity in the global morphospace

**Figure 4:**
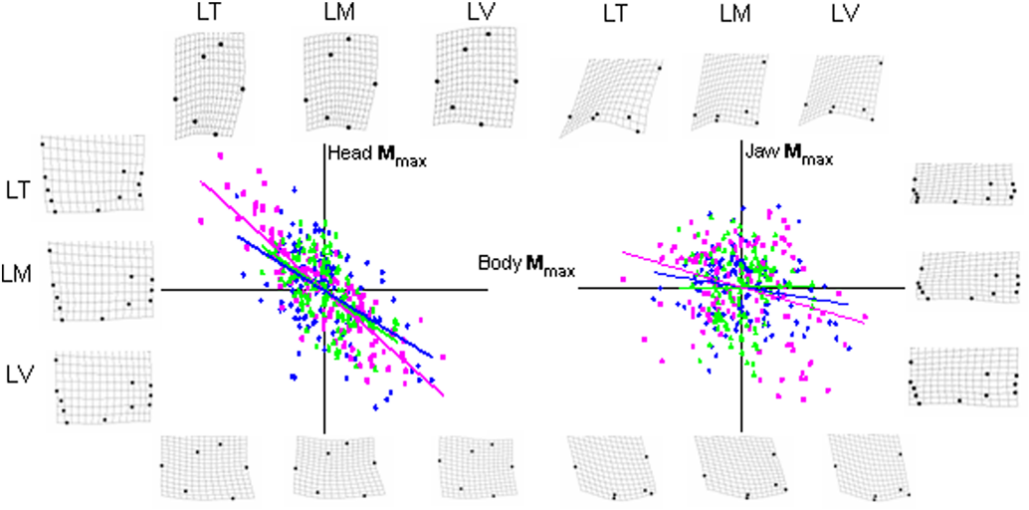
Morphological diversity in the global morphospace. Each element of shape (body, head, jaw) was analyzed separately for each assemblage (colors as in Figure 2); the three assemblages are plotted along common axes for comparison. The images show for each assemblage the shape corresponding to the most extreme positive and negative value along each M_max_ axis. See Table 2 for statistical tests of parallel divergence. Linear regression lines highlight the similar patterns of covariation between body, head and jaw shape among the assemblages.

The assemblages are diverging in parallel through the unconstrained global morphospace along every **M_max_** axis except that for total shape between LT and LV (Figure 4, Table 2). For the three elements of total shape, the assemblages are diverging in parallel except for the 2-D plane and 3-D space of jaw shape between LM and LV. The first exception is due to jaw landmarks loading more heavily on **M_max_** -total for LV than the other assemblages. This is consistent with the observation that in the common morphospace LV has its highest relative level of diversity along **M_max_**-jaw. The latter exception suggests that along minor axes of jaw shape LV is diverging differently than LM but similarly to LT. In general, only through higher dimensional spaces of total shape are the assemblages not diverging in parallel, a result of landmarks loading differently on the minor axes of the three assemblages. Patterns of morphological diversification among the shape elements appear to covary similarly in the three assemblages. For example, in all three assemblages deep bodies are associated with short “down-turned” heads associated with strong biting force, whereas elongate bodies tend to have elongate “up-turned” heads typical of planktivorous suction feeders (Figure 4).

**Table 2:** Comparison of cichlid assemblages in the global morphospace.

dimensions	total shape			body shape			head shape			jaw shape		
	t-m	t-v	m-v	t-m	t-v	m-v	t-m	t-v	m-v	t-m	t-v	m-v
**M_max_** , 90°	41.9	**78.9**	59.1	19.5	20.7	15.8	9.8	22.2	17.1	14.2	17.3	23.9
2, 127°	73.2	75.4	**57.5**	78.3	60.3	64.9	57.4	82.5	34.9	20.5	30.0	**41.9**
3, 156°	**83.3**	**81.5**	**66.7**	58.4	66.8	44.2	23.1	34.7	34.2	21.5	50.4	**54.6**
4, 180°	**107.4**	**106.2**	**95.4**	86.4	68.1	66.5	30.0	37.3	34.1	12.2	18.1	21.9

The angle between **M_max_**-axes, 2-D planes, and 3- and 4-D spaces is in bold if the two assemblages are diversifying in non-parallel directions. The first column gives the dimension of the comparison and the maximum possible angle between axes, planes or spaces.

The angles between the assemblages are consistently higher for total shape than for body, head and jaw shape (Table 2). This is particularly evident in comparisons of the **M_max_** axes. This is because partitioning total shape into contingent elements reduces the number of landmarks and morphological combinations available. As a result, the **M_max_** axes of the assemblages are more similar. Still, for total shape relatively large angles between the **M_max_** axes and the 2-D planes are not significantly different. This is because, just as in the common morphospace (Table 1), the difference between the amount of variation explained by **M_max_** and **M_2_** is less for total shape than for body, head or jaw shape. As a result, the bootstrapped confidence intervals for the first and second axes of total shape have large uncertainty and even relatively large angles are not significantly different.

### Morphological variance-covariance structures

**Figure 5:**
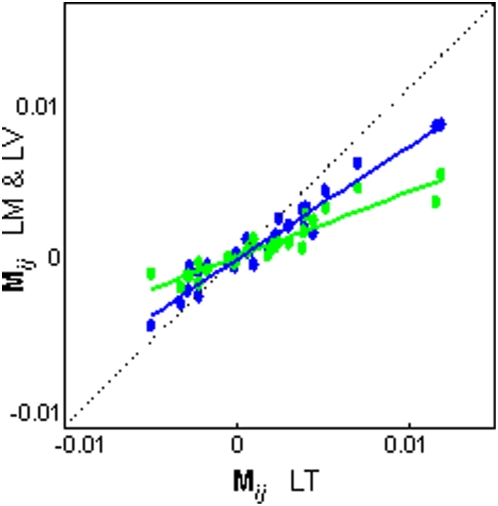
Morphological variance-covariance structures. Matrix correlation plot of the elements (*M_ij_*) from the morphological variance-covariance matrices of the three assemblages (colors as in Fig. 2). The near perfect linear relationships show the assemblages have similar variance-covariance structures across shape elements (LT-v-LM, *r* = 0.97, *P*<0.0001; LT-v-LV, *r* = 0.93, *P*<0.0001; LM-v-LV, *r* = 0.91, *P*<0.0001). The magnitudes of the matrix elements are age-ordered (linear regression slopes of LM *M_ij_* and LV *M_ij_* on LT *M_ij_*: LM = 0.67 (SE = 0.03), LV = 0.38 (SE = 0.03).

Not only are the assemblages diverging in parallel through the global morphospaces, but as Figure 4 suggests, the correlations between body, head and jaw shape are similar for each. The morphological variance-covariance matrices of the assemblages based on the **M_max_** scores of seven sub-elements have nearly identical structures (Figure 5). The slopes of LM and LV in the matrix correlation plots show that the magnitudes of the matrix elements are ranked by assemblage age, confirming the pattern of age-ordered diversity observed in the common morphospace analysis.

## Discussion

The endemic cichlids assemblages of the three African great lakes are a famous example of convergent evolution where the same sets of ecomorphs have evolved independently in each basin [16]–[18]. Despite occupying broadly overlapping areas in a common morphospace, the assemblages show consistent evidence of non-convergence, a pattern traditionally interpreted as evidence for the importance of contingency and/or chance. We first consider the possibility that this tendency toward non-convergence in fact results from deterministic evolution if differences in physical habitat and/or non-cichlid fish species composition among the lakes influence ecological opportunity and resulting patterns of morphological diversity [1]. The bathymetries of LM and LT are nearly identical; LV is larger and shallower with more demersal habitat. If the degree of non-convergence observed in the common morphospace were due to differences in physical habitat, we would expect the cichlid assemblages of LM and LT to be more similar to each other than to that of LV. Our analyses reveal no such pattern (Table 1).

The non-cichlid communities of LV and LM are similar, whereas that of LT is more speciose and contains an endemic pelagic community. If community composition of non-cichlids strongly constrains patterns of morphological diversity, the assemblages of LV and LM should be similar and more diverse than the LT assemblage. There is some evidence for the first pattern and clearly none for the second. LV and LM have the same intercept along four axes of the common morphospace, yet along no axis does either have the same intercept as LT (Table 1). Without exhaustive field studies of ecological interactions between endemic cichlids and non-endemics we cannot rule out the possibility that the observed level of non-convergence in the common morphospace results in part from deterministic evolution. However, because each lake contains the same suite of habitats and the endemic cichlid assemblages contain convergent sets of ecomorphs, we consider it unlikely that physical and biological differences among the lakes are alone responsible for the degree of non-convergence observed in the common morphspace.

Phylogenetic contingency and evolutionary chance may result in non-convergence through at least three non-exclusive mechanisms. The first invokes colonization history directly. The LT assemblage contains several phylogenetically independent radiations originating from different colonizing lineages, one of which gave rise to the haplochromine ancestors of the LM and LV radiations [19]. It is possible that through some combination of phylogenetic contingency and chance the radiations began, and remain today, centered at different areas of morphospace. The tendency for LV and LM to be centered more closely to each other than to LT in the common morphospace is consistent with this explanation. The latter two involve the effects of contingency and chance on axes of divergence through morphospace. The radiations may show evidence of non-convergence if constraints imposed by the phylogenetic history and genetic diversity of colonizing lineages have resulted in non-parallel axes of divergence through morphospace. Non-convergence could also result if adaptive radiation is simply not deterministic and, as in replicate *Escherichia coli* lineages diversifying in identical environments [5], the three cichlid assemblages are by chance ascending different ridges of the adaptive landscape (axes of morphospace), leading to different morphological solutions to similar sets of ecological opportunities [11].

Our second analysis suggests any non-convergence in the common morphospace is not the result of the assemblages diversifying along different axes through the global morphospace. Rather, the two analyses together reveal that despite differences in ecological context and phylogenetic history, and the inevitable contribution of chance that combine to produce non-convergence in a common morphospace, the cichlid assemblages of the African great lakes are diversifying in parallel through the global morphospace. Parallel divergence may be due to deterministic evolution if natural selection drives diversification along similar morphological axes in all three lakes, or phylogenetic contingency if those axes are determined by genetic constraints shared by the assemblages. The view that parallel divergence is the result of deterministic evolution rather than phylogenetic contingency is supported by the observation that the closely related and less phylogenetically diverse LV and LM radiations are not diversifying through the global morphospace along axes more similarly to each other than to the polyphyletic LT assemblage (Table 2, Figure 4). Alternatively, if the genetic/functional constraints that control axes of morphological diversification originate deeper in the cichlid phylogeny and are shared by all three assemblages, parallel divergence may reflect the role of contingency.

Our results underscore the value of comparing radiations and polyphyletic assemblages of widely different age and viewing adaptive radiation not only as an endpoint (patterns of diversity in a common morphospace) but as a process (axes of divergence through the global morphospace). Considering a celebrated example of convergent adaptive radiation highlights this point. Caribbean *Anolis* lizards have diversified on the four 10–30 myr. old islands of the Greater Antilles [3]. The radiations (some of which, like the LT assemblage, are polyphyletic) have each produced the same four ecomorphs, but phylogenetic reconstructions suggest the order in which they emerged differed between islands. Though ancestor state reconstructions are inherently uncertain [28], if we compared the radiations in a common morphospace millions of years ago, we may have found each with a different and apparently random subset of ecomorphs, concluded they were non-convergent and credited chance or contingency with trumping determinism. Similarly, though the cichlid radiations show evidence of non-convergence due to some combination of contingency, chance and ecological setting, our results show they are in fact diversifying in parallel through the global morphospace. The combined lesson from lizards and cichlids for comparative studies of adaptive radiation is that apparently idiosyncratic and non-convergent patterns of diversity may mask parallel patterns of diversification.

The relative ages of the cichlid assemblages make our results relevant to two outstanding questions about the temporal progress of adaptive radiation [1]. Disjunction between morphological and taxonomic diversity is widespread in the fossil record [29], with morphological diversity typically accumulating more rapidly and plateauing earlier than taxonomic diversity. Schluter [1] found evidence suggesting this is not the case in the extant radiations of great lake cichlids [30], lizards [3] and birds [6]. To the degree that the different cichlid assemblages represent snapshots through time of a common evolutionary process [10], we confirm and extend his result; during adaptive radiation morphological diversity accumulates continually and is unrelated to species diversity, which peaks early and declines through time. Importantly, this conclusion is unaffected by comparing the old polyphyletic assemblage of LT, consisting of several radiations, with two younger and perhaps largely monophyletic assemblages because even the youngest of the LT sub-radiations is older than those of LV and LM. Along with spider radiations on islands [10], the cichlid assemblages provide empirical support for the taxonomic “overshooting effect” [31], a temporal pattern expected if as adaptive radiation proceeds the speciation rate declines following an early burst while extinction rate remains constant. To date no species level phylogenies exist for the LV and LM radiations (except for a few small sub-clades). As molecular phylogenies improve we will be able to clarify the temporal relationship between cladogenesis and morphological diversification [32] and explore in more detail the evolution of morphological covariance structure during adaptive radiation [33].

Finally, evidence that diversity in head and jaw shape accumulates faster than in body shape supports the view that diversification during adaptive radiation proceeds at trait-dependent rates [34], [35]. We had, however, expected the opposite pattern -that body shape diversity would plateau earlier- based on the idea that radiations begin with the partitioning of physical habitat and progress by the fine scale partitioning of consumable resources [36]. Without additional radiations younger than LV we cannot rule out the possibility that body shape diversity accumulates more rapidly during the earliest stages of adaptive radiation, but the observed pattern is consistent with the long-standing hypothesis that evolutionary lability in trophic morphology has facilitated the rapid diversification in cichlid radiations [37].

Deterministic evolution, phylogenetic contingency and chance can all influence patterns of diversification during adaptive radiation. By combining traditional and new comparative approaches we have demonstrated how apparently non-convergent patterns of morphological diversity may mask parallel patterns of morphological divergence during adaptive radiation. Considering patterns of diversity in both common and global morphospaces enhances our ability to infer the roles of determinism, contingency and chance during adaptive radiation.

## Supporting Information

Table S1 (0.05 MB DOC)
