# Supplementary material for: Morphological Diversity and the Roles of Contingency, Chance and Determinism in African Cichlid Radiations
Source: PLoS One. 2009 Mar 9;4(3):e4740. doi: 10.1371/journal.pone.0004740 (PMC2648897; doi:10.1371/journal.pone.0004740)
Supplement: Table S1 — (0.05 MB DOC) [file pone.0004740.s001.doc]

**Table S1 The species/morphotypes included in the analyses**

**LAKE MALAWI LAKE TANGANYIKA LAKE VICTORIA**

Alticorpus macrocleithrum Altolamprologus compressiceps "Haplochromis" boops

Alticorpus pectinatum Asprotilapia leptura "Haplochromis" cnester

Aristochromis christyi Astatoreochromis straeleni "Haplochromis" cronus

Aulonacara guentheri Aulonocranus dewindti "Haplochromis" teunisrasi

Aulonocara macroshir Bathybates fasciatus "Haplochromis sp. "orange cinereus"

Aulonocara maylandi kandeen Bathybates ferox "Haplochromis sp. "red tridens"

Aulonocara rostrata Bathybates graueri Allochromis welcommei

Aulonocara saulosi Bathybates leo Astatotilapia barbarae

Buccochromis atritaeniatus Bathybates minor Astatotilapia brownae

Buccochromis lepturus Benthochromis tricoti Astatotilapia latifasciata

Buccochromis nototenia Boulengerochromis microlepis Astatotilapia macrops

Caprichromis liemi Callochromis macrops Astatotilapia nubila

Caprichromis orthognathus Callochromis melanostigma Astatotilapia velifer

Champsochromis caeruleus Callochromis stappersii Enterochromis 75

Champsochromis spilorhynchus Cardiopharynx schoutedeni Enterochromis cinctus

Cheilochromis euchilus Chalinochromis popelini Enterochromis erythrocephalus

Chilotilapia rhoadesii Ctenochromis horei Enterochromis sp. "kribensis"

Copadichromis eucinostomus Cunningtonia longiventralis Gaurochromis emposdisma

Copadichromis flavimanus Cyathopharynx furcifer Gaurochromis obtusidens

Copadichromis nkatae Cyphotilapia gibberosa Gaurochromis simpsoni

Copadichromis trewavasae Cyphotilapia frontosa Haplochromis lividus

Copadichromis trimaculatus Cyprichromis leptosoma Haplochromis obliquidens

Copadichromis virginalis Cyprichromis pavo Harpagochromis apogonoides

Corematodus shiranus Cyprichromis zonatus Harpagochromis cavifrons

Corematodus taeniatus Ectodus descampsii Harpagochromis diplotaenia

Ctenopharynx nitidua Enantiopus melanogenys Harpagochromis guiarti

Cyathochromis obliquidens Eretmodus cyanostictus Harpagochromis maculipinna

Cynotilapia afra Gnathochromis permaxillaris Harpagochromis martini

Cyrtocara moorii Gnathochromis pfefferi Harpagochromis michaeli

Dimidiochromis compressiceps Grammatotria lemairii Harpagochromis pachycephalus

Dimidiochromis strigatus Greenwoodochromis bellcrossi Harpagochromis plagiostoma

Diplotaxodon aeneus Ctenochromis benthicola Harpagochromis serranus

Diplotaxodon argenteus Astatotilapia burtoni Harpagochromis sp. "two- stripe yellowgreen"

Diplotaxodon eugnathus Haplotaxodon microlepsis Harpagochromis tanaos

Diplotaxodon greenwoodi Haplotaxodon trifasciatus Harpagochromis thuragnathus

Diplotaxodon limnothrissa Hemibates stenosoma Hoplotilapia retrodens

Docimodus evelynae Julidochromis marlieri Labrochromis humilior

Eclectochromis festivus Julidochromis transcriptus Labrochromis ishmaeli

Exocochromis anagenys Lamprologus callipterus Labrochromis mylodon

Fossochromis rostratus Lamprologus lemairii Labrochromis pharyngomylus

Genyochromis mento Lamprologus lemairii 2 Labrochromis teegelaari

Gephyochromis moorii Lamprologus laparogramma Lipochromis cryptodon

Astatotilapia calliptera Lamprologus ornatipinnis Lipochromis maxillaris

"Haplochromis" phenochilus Lamprologus signatus Lipochromis microdon

Hemitaeniochromis urotaenia Lepidiolamprologus attenuatus Lipochromis obesus

Hemitilapia oxyrhynchus Lepidiolamprologus elongatus Lipochromis parvidens

Iodotropheus sprengerae Lepidiolamprologus profundicola Macropleurodus bicolor

Iodotropheus stuartgranti Lestradea persipax Mbipia lutea

Labeotropheus fuelleborni Limnochromis auritus Mbipia mbipi type 1

Labeotropheus trewavasae Limnochromis staneri Mbipia mbipi type 2

Labidochromis gigas Limnotilapia dardennii Neochromis gigas

Labidochromis mylodon Lobochilotes labiatus Neochromis greenwoodi type 1

Labidochromis shiranus Microdontochromis tenuidentata Neochromis greenwoodi type 2

Lethrinops altus Neolamprologus bifasciatus Neochromis greenwoodi type 3

Lethrinops christyi Neolamprologus brichardi Neochromis omnicaeruleus blue

Lethrinops gossei Neolamprologus christyi Neochromis omnicaeruleus yellow

Lethrinops leptodon Neolamprologus crassus Neochromis rufocaudalis type 1

Lethrinops lunaris Neolamprologus cylindricus Neochromis rufocaudalis type 2

Lethrinops microdon Neolamprologus falcicula Neochromis rufocaudalis type 3

Lethrinops parvidens Neolamprologus fasciatus Paralabidochromis beadlei

Lethrinops polli Neolamprologus furcifer Paralabidochromis chilotes

Lichnochromis acuticeps Neolamprologus helianthus Paralabidochromis chromogynos

Maravichromis formosus Neolamprologus leleupi Paralabidochromis crassilabris

Maravichromis guentheri Neolamprologus longior Paralabidochromis cyaneus

Maravichromis lateristriga Neolamprologus modestus Paralabidochromis plagiodon

Maravichromis semipalatus Neolamprologus moorii Paralabidochromis sp.

Melanochromis auratus Neolamprologus mustax Paralabidochromis sp. "orange anal scraper"

Melanochromis labrosus Neolamprologus nigriventis Paralabidochromis sp. "red short snout scraper"

Melanochromis melanopterus Neolamprologus obscurus Platyaeniodus degeni

Melanochromis perspicax Neolamprologus petricola Prognathochromis sp. "black pectoral"

Pseudotropheus fainzilberi Neolamprologus pleuromaculatus Prognathochromis arcanus type 1

Mylochromis anaphyrmus Neolamprologus prochilus Prognathochromis arcanus type 2

Mylochromis balteatus Neolamprologus pulcher Prognathochromis argenteus type 1

Mylochromis ensatus Neolamprologus savoryi Prognathochromis argenteus type 2

Mylochromis ericotaenia Neolamprologus sexfasciatus Prognathochromis argenteus type 3

Mylochromis gracilis Neolamprologus splendens Prognathochromis artaxerxis

Naevochromis chrysogaster Neolamprologus tetracanthus Prognathochromis bareli

Nimbochromis linni Neolamprologus toae Prognathochromis bayoni

Nimbochromis livingstonii Neolamprologus tretocephalus Prognathochromis dentex

Nyassachromis leuciscus Neolamprologus wauthioni Prognathochromis dichrourus

Oreochromis karongae Opthalmotilapia nasuta Prognathochromis flavipinnis

Oreochromis shiranus Opthalmotiliapia boops Prognathochromis macrognathus

Otopharynx decorus Oreochromis tanganicae Prognathochromis melichrous type1

Otopharynx lithobates Paracyprichromis brieni Prognathochromis melichrous type2

Otopharynx pachycheilus Paracyprichromis nigripinnis Prognathochromis mento type 1

Otopharynx tetrastigma Perissodus eccentricus Prognathochromis mento type 2

Otopharynx walteri Perissodus microleptus Prognathochromis orange yellow small teeth sp. type 1

Pallidochromis tokolosh Petrochromis famula Prognathochromis orange yellow small teeth sp. type 2

Petrotilapia genalutea Petrochromis macrognathus Prognathochromis paraguiarti

Petrotilapia nigra Petrochromis orthognathus Prognathochromis percoides

Placidochromis electra Petrochromis polyodon Prognathochromis prognathus

Placidochromis johnstoni Plecodus elaviae Prognathochromis pyrrhopteryx

Placidochromis milomo Plecodus multidentatus Prognathochromis sp. "sharpsnout"

Protomelas annectens Plecodus paradoxus Prognathochromis sp. "smallhead"

Protomelas kirkii Plecodus straeleni Prognathochromis sp. "sowergi"

Protomelas pleurotaenia Pseudosimochromis curvifrons Prognathochromis sp. "thicklip"

Pseudotropheus ater Reganochromis calliurus Prognathochromis sp. "twenty"

Pseudotropheus barlowi Simochromis diagramma Prognathochromis worthingtonii

Pseudotropheus elongatus Simochromis marginatus Prognathochromis xenostoma

Pseudotropheus fuscoides Spathodus erythrodon Psammochromis acidens

Pseudotropheus fuscus Spathodus marlieri Psammochromis aelocephalus

Pseudotropheus greshakei Telmatochromis brichardi Psammochromis cassius

Pseudotropheus livingstoni Tangachromis dhanisi Psammochromis saxicola

Pseudotropheus longior Tanganicodus irsacae Ptyochromis granti

Pseudotropheus macrothalmus Telmatochromis temporalis Ptyochromis prodromus

Pseudotropheus minutus Telmatochromis vittatus Ptyochromis sauvagei

Pseudotropheus tropheops Trematocara variabile Ptyochromis xenognathus

Pseudotropheus williamsi Trematocara zebra Pundamilia nyererei

Pseudotropheus zebra Trematochromis marginatum Pundamilia pundamilia

Rhamphochromis brevis Trematochromis stigmaticum Pyxichromis parorthostoma

Rhamphochromis esox Triglachromis otostigma Tridontochromis chlorochrous

Rhamphochromis leptosoma Tropheus annectens Tridontochromis cryptogramma

Rhamphochromis lucius Tropheus brichardi Tridontochromis dolychorhynchus

Rhamphochromis macropthalmus Tropheus moorii Tridontochromis nanoserranus

Sciaenochromis benthicola Tropheus polli Tridontochromis plutonius

Sciaenochromis psammophilus Tylochromis polylepsis Tridontochromis tridens

Stigmatochromis pholidophorous Variabilichromis moorii Xystichromis nuchisquamulatus

Stigmatochromis woodi Xenochromis hecqui Xystichromis phytophagus

Taeniochromis holotaenia Xenotilapia boulengeri Yssichromis fusiformis

Taeniolethrinops furcicauda Xenotilapia burtoni Yssichromis laparogramma

Taeniolethrinops praeorbitalis Xenotilapia caudafasciata Yssichromis megalops

Tramitichromis brevis Xenotilapia longispinis Yssichromis piceatus

Trematocranus placodon Xenotilapia ochrogenys Yssichromis pyrrhocephalus

Tyrannochromis macrostoma Xenotilapia sima Yssichromis sp. "plumbus"

Tyrannochromis nigriventer Xenotilapia spiloptera Yssichromis sp. "supramacrops"
